# Preventing ATP Degradation by ASO-Mediated Knockdown of CD39 and CD73 Results in A2aR-Independent Rescue of T Cell Proliferation

**DOI:** 10.1016/j.omtn.2020.06.020

**Published:** 2020-06-27

**Authors:** Julia Festag, Tamara Thelemann, Monika Schell, Stefanie Raith, Sven Michel, Frank Jaschinski, Richard Klar

**Affiliations:** 1 Secarna Pharmaceuticals GmbH & Co. KG, 82152 Planegg/Martinsried, Germany

**Keywords:** adenosine axis, ATP, CD39, CD73, ectonucleotidase, A2a receptor, antisense oligonucleotide, ASO, immunotherapy, tumor microenvironment

## Abstract

The adenosine axis contributes to the suppression of antitumor immune responses. The ectonucleotidase CD39 degrades extracellular adenosine triphosphate (ATP) to adenosine monophosphate (AMP), which is degraded to adenosine by CD73. Adenosine binds to, e.g., the A2a receptor (A2aR), which reportedly suppresses effector immune cells. We investigated effects of ATP, AMP, and adenosine analogs on T cell proliferation, apoptosis, and proinflammatory cytokine secretion. CD39 and CD73 expression were suppressed using antisense oligonucleotides (ASOs), and A2aR was blocked using small molecules. Addition of ATP to T cells reduced proliferation and induced apoptosis. Intriguingly, those effects were reverted by suppression of CD39 and/or CD73 expression but not A2aR inhibition. Adenosine analogs did not suppress proliferation but inhibited secretion of proinflammatory cytokines. Here, we suggest that suppression of T cell proliferation is not directly mediated by A2aR but by intracellular downstream metabolites of adenosine, as blockade of the equilibrative nucleoside transporter (ENT) or adenosine kinase rescued proliferation and prevented induction of apoptosis. In conclusion, adenosine might primarily affect cytokine secretion directly via adenosine receptors, whereas adenosine metabolites might impair T cell proliferation and induce apoptosis. Therefore, inhibition of CD39 and/or CD73 has evident advantages over A2aR blockade to fully revert suppression of antitumor immune responses by the adenosine axis.

## Graphical Abstract

**Figure:**
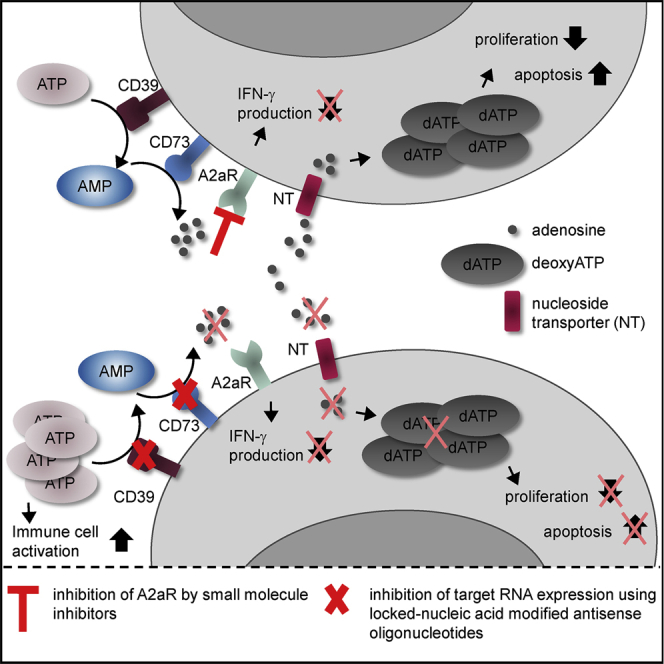

## Teaser

The unleashing of antitumor immune responses by targeting the purine metabolism has emerged as a promising strategy in cancer immunotherapy. Festag and colleagues targeted different components of the purine metabolism using antisense oligonucleotides and small molecules and could show that T cell proliferation and cytokine release are differentially suppressed by distinct purine metabolites.

## Introduction

Tumors can escape immune recognition and destruction by a plethora of immunosuppressive mechanisms. The unleashing of tumor-specific immune responses by blocking immunosuppressive pathways has emerged as a promising treatment option in the last years. Despite the remarkable success of blocking immune checkpoints, like programmed death 1 (PD-1), using monoclonal antibodies, only a minority of patients benefit from the currently available immunotherapies.^1^ The adenosine axis has emerged as a promising therapeutic target to enhance antitumor immunity. Degradation of extracellular immune-stimulating adenosine triphosphate (ATP) by the ectonucleotidase CD39 to adenosine monophosphate (AMP) is followed by generation of immunosuppressive adenosine by CD73. Adenosine binds to adenosine receptors, e.g., the A2a receptor (A2aR) or with lower affinity to the A2b receptor (A2bR), which increases intracellular cyclic AMP (cAMP) levels, resulting in suppression of immune cell function.^2^

ATP is released from dying cells and exhibits antitumor effects in two ways: (1) immune stimulation through recruitment and activation of dendritic cells and macrophages, which in turn leads to activation of T and natural killer (NK) cells;^3^, ^4^, ^5^ (2) specific inhibition of tumor cell proliferation and induction of tumor cell death.^6^^,^^7^ CD39 is expressed on a wide range of immune cells, including monocytes, macrophages, dendritic cells, B, T, and NK cells, as well as on endothelial cells.^8^ Moreover, CD39 can be highly expressed by tumor cells themselves, for example, in lung, kidney, testicular, and thyroid cancer, as well as lymphoma and melanoma.^9^ The targeting of CD39 function with antibodies or suppression of its expression with locked nucleic acid (LNA)-modified antisense oligonucleotides (ASOs) has shown promising results in preclinical studies, especially in combination with either chemotherapy or PD-1 inhibition.^5^^,^^10^^,^^11^ Extracellular AMP can also be generated independent from CD39 via CD38, resulting in generation of immunosuppressive adenosine by CD73, which is also widely expressed on immune cells and tumor cells.^12^ Besides CD39 and CD73, the A2aR represents a further option to interfere with the adenosine axis. The A2aR is expressed in T cells and is induced upon T cell activation.^13^^,^^14^ Antitumor effects revealing the efficacy of inhibiting CD73 or the A2aR by antibodies or small molecule inhibitors have been demonstrated,^15^, ^16^, ^17^, ^18^, ^19^, ^20^ and currently, CD73 and A2aR blockade is being investigated in more than 20 clinical trials (phase I or II).^2^^,^^21^ A CD39 antibody (TTX-030)^5^ is clinically evaluated in combination with PD-1 inhibition or chemotherapy. This highlights the promise of therapeutically interfering with the adenosine axis in order to improve the efficacy of cancer immunotherapies.

We targeted the expression of CD39 and CD73 with LNA-modified ASOs with a fully phosphorothioated backbone that protects the ASOs from degradation by nucleases.^22^ The LNA-modified flanks further increase stability and lead to increased target affinity, whereas the central unmodified “gap” allows for recruitment of RNase H, which in turn, cleaves the RNA upon binding of the ASO to its target. Unlike earlier chemical modifications, LNA gapmers exert target suppression without the use of a transfection reagent (gymnosis).^23^^,^^24^ Notably, target knockdown *in vivo* can be achieved in several tissues, including tumor cells, after systemic administration without the need for a delivery reagent.^10^^,^^24^

Here, we demonstrate that treatment of human T cells with LNA-modified ASOs specific for human CD39 and CD73 results in potent target knockdown *in vitro* without the use of a transfection reagent. Moreover, downregulation of CD39 and/or CD73 in T cells by ASO treatment, but not A2aR inhibition by small molecules, reverted the inhibition of T cell proliferation and prevented the induction of apoptosis induced by ATP degradation products. Strikingly, adenosine analogs did not suppress T cell proliferation but decreased production of proinflammatory cytokines by activated T cells, revealing that different components of the adenosine axis might be involved in suppression of production of proinflammatory cytokines and proliferation of T cells. We show that a microenvironmental factor produced by ATP degradation, other than adenosine, is responsible for the antiproliferative effect. In fact, the blocking of the equilibrative nucleoside transporter (ENT), which transports nucleoside substrates, like adenosine, into cells, or the adenosine kinase (AK), which mediates the formation of deoxyATP (dATP), completely reverts the antiproliferative effect of ATP degradation. This is probably caused by preventing the accumulation of dATP, highlighting the advantage of inhibition of CD39 and CD73 that act upstream of adenosine.

## Results

### CD39 and CD73 Expression Is Inhibited in Human T Cells after CD39- and/or CD73-Specific ASO Treatment

**Figure 1:**
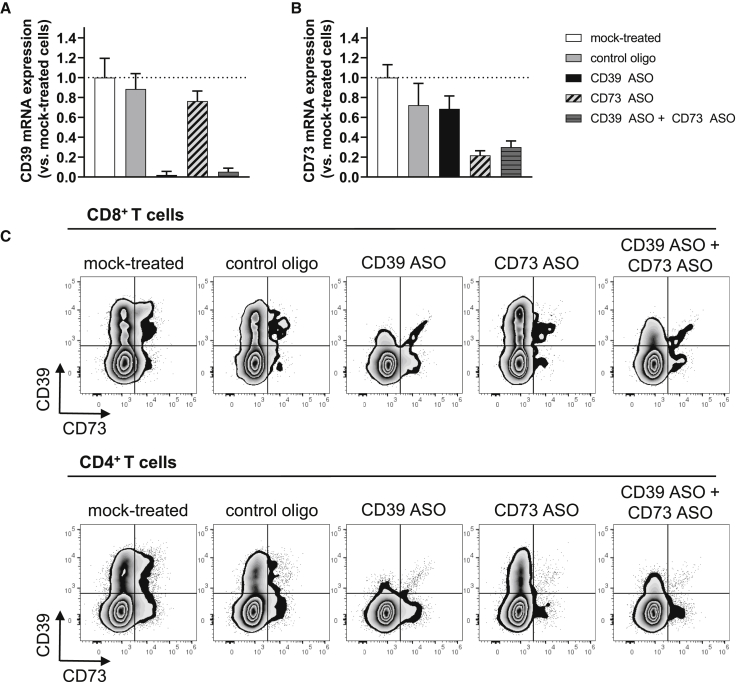
Efficacy of CD39- and CD73-Specific ASOs in Human T Cells Human T cells were activated with anti-CD2/CD3/CD28 tetrameric antibody complexes and treated with 5 μM of the respective oligonucleotide (or 2.5 μM in the case of the CD39 ASO + CD73 ASO condition) without using a transfection reagent. As mock control, cells were not treated with ASO. (A and B) After 3 days of treatment, CD39, CD73, and HPRT1 mRNA levels were determined in cell lysates. CD39 (A) and CD73 (B) values were normalized to HPRT1 expression values and are displayed relative to mock-treated control (set as 1). The mean + SD from three technical replicates is depicted. Representative results from two independent experiments are shown. (C) After 5 days of ASO treatment, CD39 and CD73 protein expression was analyzed by flow cytometry. Representative results from three technical replicates and three independent experiments are shown.

We first determined the protein expression levels of CD39, CD73, the A2aR, and the A2bR on human T cells to ensure that all components of the canonical adenosine axis were expressed in our experimental system. On day 3 after T cell activation, CD39, CD73, as well as the A2aR and the A2bR were expressed on CD8^+^ and CD4^+^ T cells. The expression levels varied, comparing CD8^+^ T cells to CD4^+^ T cells, with CD73 being highly expressed on CD8^+^ T cells, CD39 being mainly expressed on CD8^+^ T cells, and the A2aR, as well as the A2bR, expressed on CD4^+^ T cells to a higher degree (Figures S1A and S1B). As CD39 is highly expressed on regulatory T cells (T_regs_),^25^ we evaluated if the small population of CD4^+^ T cells that expressed CD39 could be identified as T_regs_. We found that approximately 50% of CD4^+^ CD39^+^ cells were T_regs_, characterized by the expression of CD25 and FoxP3 (Figures S1C and S1D). Next, we investigated the effects of CD39- and/or CD73-specific ASOs on CD39 and CD73 expression in human T cells. Therefore, T cells were activated and treated with the respective ASOs without the use of a transfection reagent, and CD39 and CD73 mRNA expression was analyzed 3 days later (Figures 1A and 1B). Treatment with the control oligo that has no sequence complementarity to any human or mouse RNA had no major effect on CD39 and CD73 mRNA levels as compared to mock-treated cells. In contrast, CD39 mRNA expression was reduced by 98% if cells were treated with 5 μM CD39 ASO and more than 95% if T cells were treated with a combination of 2.5 μM CD39 ASO and 2.5 μM CD73 ASO (Figure 1A). T cells treated with the CD73 ASO (Figure 1B) or the combination of CD39 and CD73 ASO expressed approximately 70% less CD73 mRNA compared to mock-treated cells. Moreover, CD39 and CD73 protein expression was determined by flow cytometry on day 5 after the start of treatment (Figure 1C). CD39 expression was greatly reduced in CD8^+^ as well as in CD4^+^ T cells that had been treated with CD39 ASO. Similar effects were observed for CD73 expression, although overall CD73 expression was lower as compared to CD39 expression 5 days after T cell activation and start of ASO treatment. Again, treatment with the control oligo had only minor effects on CD39 or CD73 protein expression in CD8^+^ and CD4^+^ T cells (Figure 1C). Thus, CD39 and CD73 mRNA and protein expression can be potently and specifically reduced in activated human T cells by LNA-modified ASOs without the need for a transfection reagent.

### CD39 and/or CD73 ASO Treatment Rescues T Cells from ATP-Induced Inhibition of Proliferation

**Figure 2:**
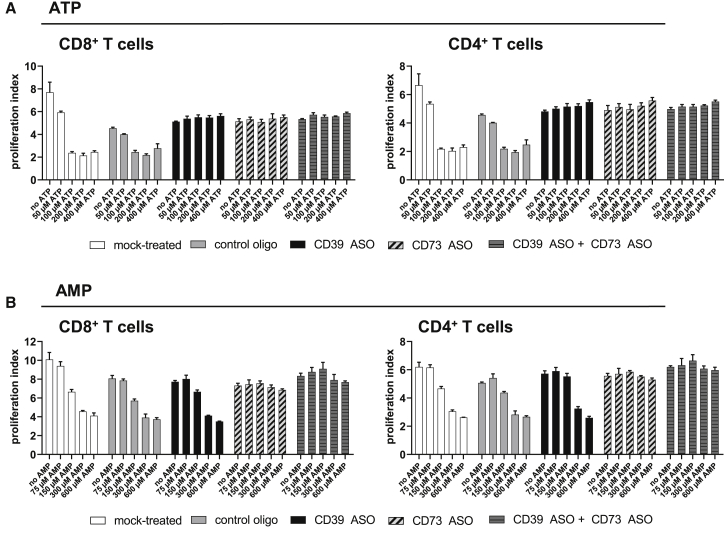
Effect of CD39- and CD73-Specific ASOs on T Cell Proliferation in the Presence or Absence of Extracellular ATP/AMP Human T cells were labeled with a proliferation dye, activated with anti-CD2/CD3/CD28 tetrameric antibody complexes, and treated with 5 μM of respective ASOs (or 2.5 μM in the case of the CD39 ASO + CD73 ASO condition) without using a transfection reagent. As mock control, cells were not treated with an ASO. On days 3 and 4, medium was supplemented with different doses of ATP (A) or AMP (B). Proliferation of T cells was analyzed by flow cytometry on day 5, and proliferation indices of CD8^+^ and CD4^+^ T cells were calculated. The mean + SD of three technical replicates is depicted. Representative results from two independent experiments are shown.

We have previously shown that CD39 ASO treatment of CD8^+^ T cells is able to revert the inhibitory effect of degradation products derived from extracellular ATP on CD8^+^ T cell proliferation.^10^ In the present study, CD3^+^ T cells were activated and exposed to ASOs for 3 days before the cells were cultured in the presence of extracellular ATP for 2 more days. Rescue of T cell proliferation in CD39-ASO-treated cells was seen for CD8^+^ as well as CD4^+^ T cells up to a concentration of 400 μM of extracellular ATP (Figure 2A). Furthermore, also T cells that had been treated with CD73 ASO were protected from the inhibition of proliferation induced by ATP degradation products. This finding suggests that not AMP but a downstream degradation product of AMP is responsible for the reduced number of T cells in mock- and control oligo-treated samples in the presence of increasing concentrations of extracellular ATP (Figure 2A). To further strengthen this hypothesis, we next examined the effect of extracellular AMP on T cell proliferation (Figure 2B). AMP supplementation reduced proliferation of CD8^+^ and CD4^+^ T cells in mock- and control oligo-treated samples. Moreover, T cells that had been treated with CD39 ASO only were not protected from the inhibition of proliferation induced by AMP degradation products, whereas T cell proliferation was not decreased in samples that had been subjected to CD73 ASO ± CD39 ASO at extracellular AMP concentrations up to 600 μM (Figure 2B). Taken together, inhibition of ATP degradation by inhibiting CD39 and/or CD73 expression, respectively, reverts the suppression of T cell proliferation.

### A2aR Inhibition Does Not Prevent ATP Degradation-Induced Reduction of T Cell Proliferation

**Figure 3:**
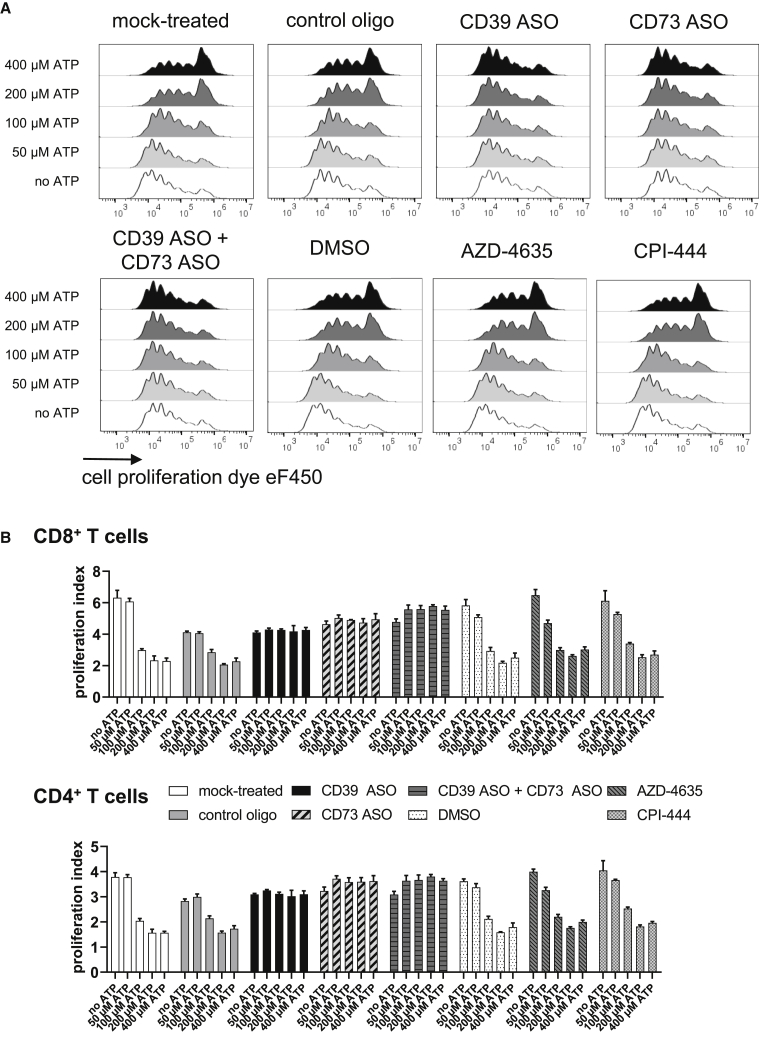
Impact of A2aR Inhibition on T Cell Proliferation in the Presence or Absence of Extracellular ATP Human T cells were labeled with a proliferation dye, activated with anti-CD2/CD3/CD28 tetrameric antibody complexes, and treated with 5 μM of respective ASOs (or 2.5 μM in the case of the CD39 ASO + CD73 ASO condition) without using a transfection reagent. As mock control, cells were not treated with an ASO. On days 3 and 4, 10 μM of A2a receptor inhibitors (A2aRIs) AZD-4635 or CPI-444, dissolved in DMSO, or DMSO and different doses of ATP, was added to the cells. Proliferation of T cells was analyzed by flow cytometry on day 5 (A), and proliferation indices of CD8^+^ and CD4^+^ T cells were calculated (B). (A) Representative results from three technical replicates and two independent experiments are shown. (B) The mean + SD from three technical replicates is depicted. Representative results from two independent experiments are shown.

A2aR inhibitors (A2aRIs), as AZD-4635 or CPI-444, counteract immunosuppression mediated by the ATP and AMP degradation product adenosine.^17^, ^18^, ^19^, ^20^, ^21^ We could confirm that the A2aRIs AZD-4635 and CPI-444 restored interferon (IFN)-γ production that was suppressed by the stable adenosine analog 5′-(N-ethylcarboxamido) adenosine (NECA) in peripheral blood mononuclear cells (PBMCs) (Figure S2). Next, we assessed the effect of A2aRIs on inhibition of proliferation induced by ATP degradation products. Strikingly, increasing concentrations of extracellular ATP led to decreased proliferation of CD8^+^ and CD4^+^ T cells in A2aRI-treated samples comparable to DMSO-treated cells (Figures 3A and 3B). As observed before, CD39- and/or CD73 ASO-treated T cells were protected from this antiproliferative effect induced by ATP degradation products. In summary, A2aRIs restored IFN-γ production of activated PBMCs in the presence of NECA but could not prevent reduction of T cell proliferation induced by ATP degradation products.

### Adenosine Analogs NECA, CGS 21680, and 2-Chloro-Adenosine (CADO) Do Not Inhibit T Cell Proliferation

**Figure 4:**
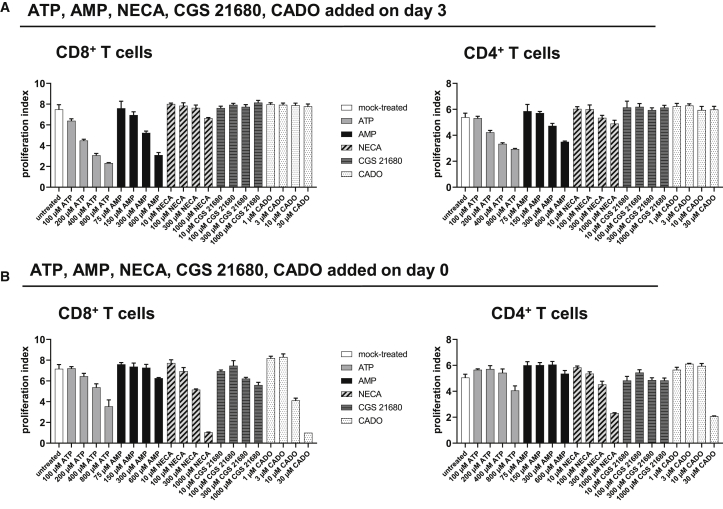
Influence of Extracellular ATP, AMP, and the Adenosine Analogs NECA, CGS 21680, and CADO on T Cell Proliferation (A and B) Human T cells were labeled with a proliferation dye, activated with anti-CD2/CD3/CD28 tetrameric antibody complexes, and treated with ATP, AMP, or the adenosine analogs NECA, CGS 21680, or CADO at the indicated concentrations on day 3 (A) or day 0 (B). As mock control, cells were not treated with ATP, AMP, NECA, CGS 21680, or CADO. Proliferation of T cells was analyzed by flow cytometry on day 5, and proliferation indices of CD8^+^ and CD4^+^ T cells were calculated. The mean + SD of three technical replicates is depicted. Representative results from two independent experiments are shown.

We next assessed whether the adenosine analogs NECA, CGS 21680, and CADO had an inhibitory effect on T cell proliferation. To this end, T cells were activated for 3 days as before, and medium was supplemented with increasing concentrations of ATP, AMP, NECA, CGS 21680, or CADO. 2 days later, the proliferation indices were determined. Whereas extracellular ATP and AMP dose dependently reduced proliferation of CD8^+^ and CD4^+^ T cells, adenosine analogs NECA, CGS 21680, and CADO had no impact on T cell proliferation, even at a concentration of 1,000 μM (Figure 4A). As an inhibitory effect of CGS 21680 on mouse CD8^+^ T cell proliferation has been reported when the compound was administered on day 0,^26^ we performed the same assay but cultured the cells in the presence of ATP, AMP, NECA, CGS 21680, and CADO from day 0 (Figure 4B). Proliferation of both CD8^+^ and CD4^+^ T cells was decreased with increasing concentrations of extracellular ATP and AMP, whereby the effect of ATP and AMP was less pronounced compared to administration of the compound on day 3. Again, addition of CGS 21680 had no impact on T cell numbers, whereas NECA and CADO led to a decrease in T cell proliferation at high concentrations (Figure 4B). To sum up, A2aR signaling seems not to be the main factor that is responsible for the ATP- and AMP-induced inhibition of T cell proliferation, although the A2aR is highly expressed on activated T cells (see Figure S1).

### ATP and AMP or Their Degradation Products and Adenosine Analogs Suppress Proinflammatory Cytokine Production of Activated T Cells

**Figure 5:**
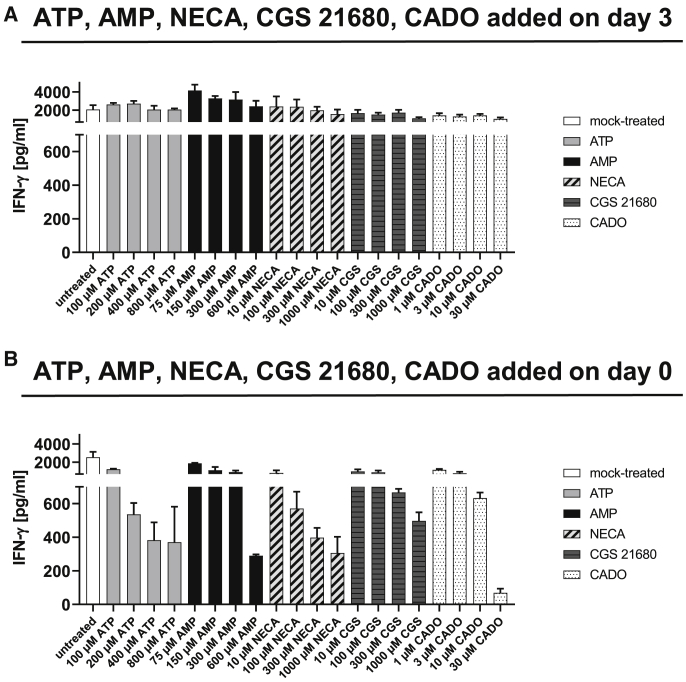
IFN-γ Concentration in T Cell Cultures Supplemented with Extracellular ATP, AMP, and the Adenosine Analogs NECA, CGS 21680, or CADO Human T cells were labeled with a proliferation dye, activated with anti-CD2/CD3/CD28 tetrameric antibody complexes, and treated with ATP, AMP, or the adenosine analogs NECA, CGS 21680, or CADO at the indicated concentrations on day 3 (A) or day 0 (B). As mock control, cells were not treated with ATP, AMP, or an adenosine analog. IFN-γ concentrations in supernatants were analyzed on day 5.

The culturing of PBMCs with NECA led to a decrease in IFN-γ production (Figure S2). As we did not observe an effect of adenosine analogs on T cell proliferation, we analyzed IFN-γ levels in the supernatants of those experiments (shown in Figures 4A and 4B). When ATP, AMP, or adenosine analogs were added on day 3 after T cell activation, the concentration of IFN-γ did not differ in any condition (Figure 5A). When we added ATP, AMP, NECA, CGS 21680, or CADO to the T cell cultures on day 0, we observed a dose-dependent reduction of IFN-γ levels (Figure 5B). To assess whether addition of ATP, AMP, and adenosine analogs did not reduce IFN-γ production when applied on day 3 or if the effect was masked by cytokines released into supernatants before addition of the compounds, we performed an intracellular cytokine staining (ICS) 2 days after adding the compounds to the T cell cultures. ATP, AMP as well as adenosine analogs reduced the frequency of CD8^+^ IFN-γ^+^ T cells (Figure S3). Moreover, the frequency of tumor necrosis factor alpha (TNF-α)^+^ CD8^+^ T cells as well as interleukin (IL)-2^+^ CD8^+^ T cells was also reduced (Figure S3). Consequently, signaling through adenosine receptors might not influence proliferation of T cells but inhibits secretion of the proinflammatory cytokines IFN-γ, TNF-α, and IL-2.

### Accumulation of an Adenosine Metabolite Is Responsible for Suppression of T Cell Proliferation

**Figure 6:**
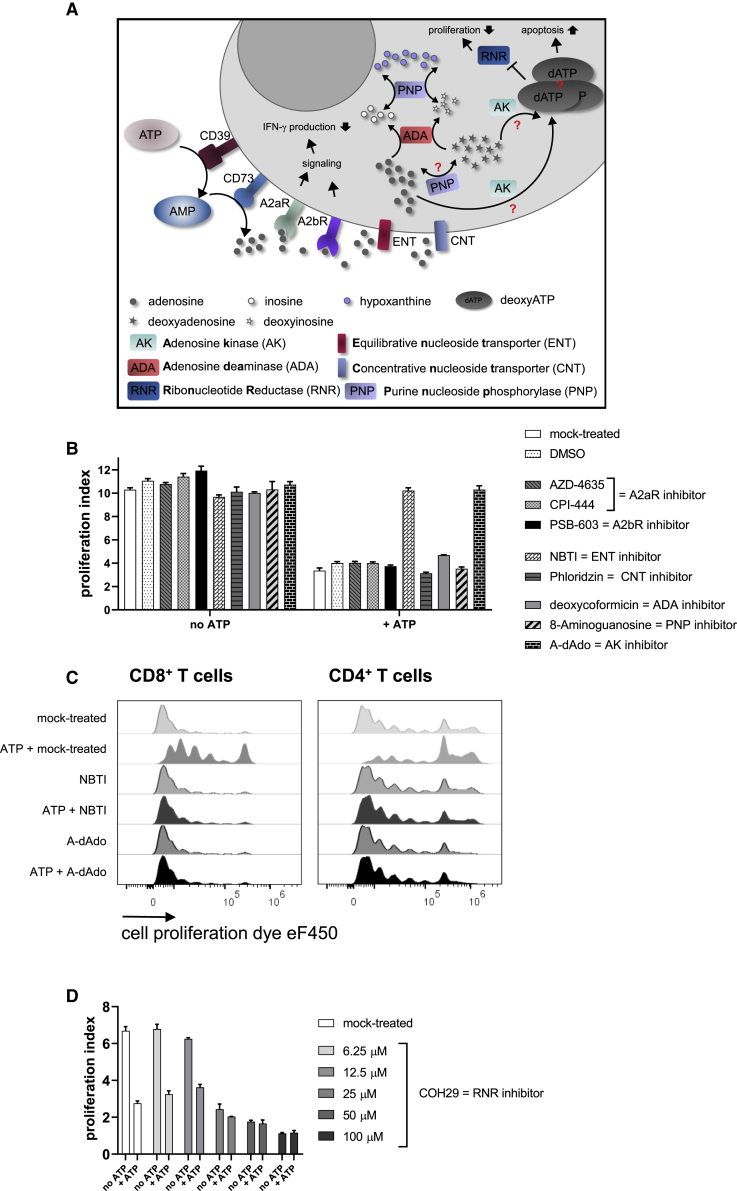
Influence of Inhibition of Different Components of the Purine Metabolism on T Cell Proliferation in the Presence of Extracellular ATP (A) Schematic illustration of the different components inhibited. (B–D) Human T cells were labeled with a proliferation dye and activated with anti-CD2/CD3/CD28 tetrameric antibody complexes. On day 3, (B) A2aR inhibitors AZD-4635 or CPI-444 (10 μM), A2bR inhibitor PSB-603 (10 μM), ENT inhibitor NBTI (20 μM), CNT inhibitor Phloridzin (20 μM), ADA inhibitor 2′-deoxycoformycin (dCF) (10 μM), PNP inhibitor 8-aminoguanosin (100 μM), AK inhibitor A-dAdo (10 μM), or (D) RNR inhibitor COH29 (at indicated concentrations) and (B–D) 400 μM ATP were added to the cells. Proliferation of T cells was analyzed by flow cytometry on day 5, and proliferation indices were calculated (B and D). (C) Proliferation of mock-treated cells and NBTI- or A-dAdo-treated cells in the absence or presence of ATP. (B and D) The mean + SD from three technical replicates is depicted. Representative results from three independent experiments are shown. (C) Representative results from three technical replicates and three independent experiments are shown.

In order to further narrow down the cause for inhibition of T cell proliferation mediated by ATP degradation products, we additionally inhibited different components of the purine metabolism (schematic illustration in Figure 6A). Next to the A2aR, we also investigated the effect of inhibitors of the A2bR and of nucleoside transporters (ENT and concentrative nucleoside transporter [CNT]). Furthermore, we inhibited the adenosine deaminase (ADA), which is responsible for the conversion of adenosine (deoxyadenosine) to inosine (deoxyinosine), and tested inhibitors of the purine nucleoside phosphorylase (PNP) that metabolizes inosine and deoxyinosine to hypoxanthine. Moreover, deoxyadenosine can be generated from adenosine by PNP with adenine as an intermediate. Finally, we blocked the AK that mediates the formation of ATP from adenosine but also uses deoxyadenosine as substrate, leading to the generation of dATP.^27^^,^^28^ Upon addition of extracellular ATP, T cell proliferation was completely rescued when the transport of adenosine through the ENT transporter was inhibited by S-(4-nitrobenzyl)-6-thioinosine (NBTI) and when 5′-amino-5′deoxyadenosine (A-dAdo), an inhibitor of the AK, was present (Figures 6B and 6C). All other inhibitors, including the PNP inhibitor 8-aminoguanosine, had no effect regarding rescue of T cell proliferation. To further evaluate the potential role of PNP in ATP-induced blockade of proliferation, an additional compound with PNP inhibitory function (9-deazaguanine) was tested. The same result as obtained with 8-aminoguanosine was observed (Figure S4). Individuals suffering from ADA deficiency are immunodeficient, and this has not been linked to enhanced A2aR signaling but to deoxyadenosine that is phosphorylated to dATP.^29^, ^30^, ^31^ Accumulation of dATP inhibits the ribonucleotide reductase (RNR) and consequently, blocks DNA synthesis, which is believed to be the cause for the inability of cells to divide. In order to evaluate whether inhibition of the RNR would have the same effect as observed upon addition of extracellular ATP, we inhibited the RNR with COH29.^32^ Addition of COH29 dose dependently inhibited proliferation of T cells, also in the absence of ATP, at doses higher than 12.5 μM (Figure 6D), indicating that blockade of the RNR could be one reason for the observed antiproliferative effect of ATP degradation products.

### CD39 and/or CD73 ASO Treatment, but Not A2aR Blockade, Prevents Induction of Apoptosis by ATP Degradation Products in Activated T Cells

**Figure 7:**
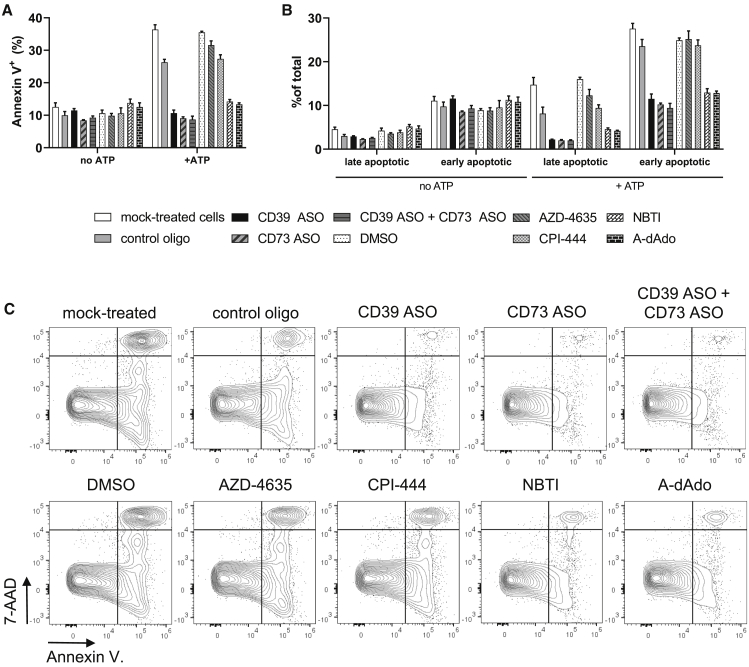
Induction of Apoptosis in the Presence or Absence of Extracellular ATP Human T cells were labeled with a proliferation dye, activated with anti-CD2/CD3/CD28 tetrameric antibody complexes, and treated with 5 μM of respective ASOs (or 2.5 μM in the case of the CD39 ASO + CD73 ASO condition) without using a transfection reagent. As mock control, cells were not treated with an ASO. On day 3, A2aRI AZD-4635 or CPI-444 (10 μM), ENT inhibitor NBTI (20 μM), AK inhibitor A-dAdo (10 μM), or DMSO and 400 μM ATP were added to the cells. Induction of apoptosis was analyzed by flow cytometry on day 5. (A) Frequency of annexin V^+^ cells. (B) Frequency of late apoptotic (7-AAD^+^ annexin V^+^) and early apoptotic (7-AAD^−^ annexin V^+^) cells. (C) Representative plots for 7-AAD and annexin V staining in the presence of extracellular ATP. (A and B) The mean + SD from three technical replicates is depicted. Representative results from three independent experiments are shown. (C) Representative results from three technical replicates and three independent experiments are shown.

Accumulation of intracellular dATP is known to induce mitochondria-dependent apoptosis.^33^, ^34^, ^35^ We therefore investigated whether addition of extracellular ATP and the proposed accumulation of dATP, originating from adenosine generated by CD39 and CD73 enzymatic activity, would not only lead to decreased proliferation but also to induction of apoptosis. Thus, activated T cells were cultured with extracellular ATP, and an annexin V staining was performed to identify apoptotic cells 2 days later. Addition of ATP led to an increased frequency of annexin V^+^ cells (Figure 7A). We further analyzed the frequency of late apoptotic (7-aminoactinomycin D [7-AAD]^+^ annexin V^+^) and early apoptotic (7-AAD^−^ annexin V^+^) cells and found that the frequency of both populations was enhanced in the presence of extracellular ATP (Figures 7B and 7C). T cells treated with CD39 and/or CD73 ASO, as well as with the ENT inhibitor NBTI or the AK inhibitor A-dAdo, were completely protected from increased induction of apoptosis (Figure 7). In strong contrast, inhibition of the A2aR with AZD-4635 and CPI-444 had no protective effect. Consequently, degradation of ATP results in the generation of adenosine that is not only affecting T cell activity through A2aR signaling but also through A2aR-independent effects, possibly through its conversion to immunosuppressive dATP.

## Discussion

Improvement of currently available immunotherapies is of high medical need, as only a minority of patients benefits from the currently available treatment options. Patients either do not respond to checkpoint blockade or experience a relapse after initial response.^1^ The adenosine axis is increasingly recognized as one of the most important immunosuppressive pathways in immuno-oncology, and the combining of agents that reduce the level of immunosuppressive adenosine with currently available immunotherapies or chemotherapies is under clinical investigation at the moment.^2^

Here, we target the expression of the ectonucleotidases CD39 and CD73 by ASOs and demonstrate that T cells treated with CD39- or CD73-specific ASOs are protected from the inhibition of proliferation induced by ATP degradation products. We observed that ASO-mediated knockdown of CD39 and/or CD73 rescued T cells from ATP-induced suppression of proliferation up to a concentration of 400 μM ATP, which is a relevant concentration measured at tumor sites as a source of immunosuppressive adenosine.^36^ Moreover, we demonstrate that addition of extracellular AMP also causes inhibition of T cell proliferation, and inhibition of CD73 expression by ASOs was able to revert this suppression. This finding suggests that a downstream degradation product of AMP is responsible for suppression of T cell proliferation. Interestingly, A2aR and A2bR blockade did not preserve T cells from the suppression of T cell expansion induced by ATP degradation products. Very importantly, adenosine analogs did not affect T cell proliferation, arguing for a distinct mechanism from adenosine receptor signaling being responsible for suppression of T cell proliferation. In contrast, adenosine analogs potently inhibited secretion of the proinflammatory cytokine IFN-γ when added to T cell cultures on day 0, suggesting that ATP degradation product-mediated suppression of proliferation is uncoupled from suppression of IFN-γ production. We did not observe a difference in IFN-γ concentrations in supernatants of cultures in which ATP, AMP, or adenosine analogs were added to the cultures on day 3 after activation. This could be explained by the fact that A2aR signaling inhibits IFN-γ production^37^ but interferes only with early T cell receptor (TCR) signaling events, and the effects are lost 3 days after activation, as reported for mouse T cells.^26^ Another explanation could be that the inhibitory effect of ATP, AMP, or adenosine analogs could have been masked by cytokines released into supernatants before addition of the compounds. In fact, an ICS in T cells on day 5 after activation that had been treated with ATP, AMP, or adenosine analogs on day 3 after activation revealed a reduction in the frequencies of IFN-γ^+^, TNF-α^+^, as well as IL-2^+^ CD8^+^ T cells. Those findings argue for proliferation being regulated by a different component of the adenosine axis as compared to proinflammatory cytokine production.

Inhibition of T cell proliferation induced by ATP degradation products was shown to be reversed by the addition of blocking antibodies targeting CD39 or CD73.^11^ The authors claim that adenosine is responsible for this effect, but this has not been demonstrated directly, and potentially, another degradation product of ATP might be responsible for suppressing T cell proliferation, as observed in our study. In fact, it has been proposed earlier that inhibition of lymphocyte proliferation by selective adenosine receptor agonists is mediated independent from the adenosine receptor.^38^ In accordance with our data, a similar result was observed in a study using mouse T cells in which A2aR signaling had strong effects on cytotoxicity and IFN-γ production of T cells, but CD8^+^ as well as CD4^+^ T cells were able to proliferate well in the presence of A2aR agonists.^39^ In line with this, in a study combining a dendritic cell-based therapeutic cancer vaccine with A2aR inhibition or a CD73 inhibitor, it was reported that A2aR inhibition did not enhance proliferation, whereas CD73 inhibition did.^40^ Additionally, in a mouse model of B cell lymphoma, a complete knockout of CD39 in T cells was able to overcome the inhibition of expansion by different purines, whereas a complete knockout of the A2aR in T cells had only little impact on T cell proliferation.^41^ Therefore, A2aR signaling likely does not completely suppress T cell function and rather has an inhibitory effect on cytokine production than on proliferation.

It has been demonstrated that proliferation of T cells was affected by addition of adenosine (100 μM) and NECA (10 μM) in coculture with ovarian cancer cells^42^^,^^43^ or U87 glioma cells.^44^ It could be speculated that the observed antiproliferative effect in these experiments was not directly exerted by the supplemented adenosine but by ATP released by the tumor cells. Moreover, an inhibitory effect of the adenosine analog CGS 21680 (1 μM) on mouse T cell proliferation has been demonstrated; however, the effect on IFN-γ was more pronounced (25% reduction in proliferation, 75% reduction of IFN-γ concentration).^26^ Furthermore, the antiproliferative effect was lost when CGS 21680 was applied 24 h after activation. This is why the authors conclude that A2aR signaling relies on blockade of early TCR signaling events, and these effects are lost once TCR signals have been transduced.^26^ However, we observed a stronger inhibitory effect on T cell proliferation when T cells were exposed to ATP and AMP degradation products from day 3 compared to day 0 after activation. This argues again for an alternative factor than adenosine and a pathway other than A2aR signaling being responsible for inhibition of proliferation, whereas A2aR signaling has a strong impact on IFN-γ production.

ADA converts adenosine to inosine and has a second naturally occurring substrate, namely deoxyadenosine, which is converted to deoxyinosine. Deoxyadenosine can be generated from adenosine by PNP. ADA deficiency is associated with severe combined immunodeficiency (SCID). A feasible explanation could be that adenosine accumulation and subsequent A2aR signaling are responsible for the observed immunodeficiency. However, it has been demonstrated that the substrate deoxyadenosine—more precisely, the accumulation of dATP—mediates the immune toxicity. Mechanistically, dATP inhibits RNR, which is responsible for the generation of deoxynucleotides, thereby preventing DNA synthesis. That, in turn, leads to the inability of cells to divide and proliferate.^29^, ^30^, ^31^ Our results demonstrate that degradation of extracellular ATP or AMP by ectonucleotidases might, besides the generation of adenosine, also lead to the accumulation of other purine metabolites, including dATP, which could suppress the proliferation of activated human T cells. To further investigate the mechanism of purine-mediated suppression of T cell proliferation, we tested inhibitors of different components of the purine metabolism pathway and identified ENTs, which transport adenosine into the cell, and the AK as important players. Inhibition of CNTs did not rescue T cells from the inhibition of proliferation by ATP degradation products, arguing for ENTs being primarily responsible for adenosine transport in primary human T cells. Treatment with 8-aminoguanosine, a published inhibitor of PNP,^45^ also did not protect T cells from the antiproliferative effect of extracellular ATP, although this would be expected, as the conversion of adenosine to deoxyadenosine would be blocked. Those results have been confirmed using a second PNP inhibitor. One could hypothesize that dATP is formed by an alternative pathway, not from deoxyadenosine, deoxyAMP, and deoxyADP but from adenosine with AMP, ADP, and deoxyADP as intermediates. Hereby, the RNR would be responsible for the conversion of ADP to deoxyADP.^46^ Thus, PNP would not be the rate-limiting step in the generation of dATP. It could be assumed that inhibition of ADA exacerbates the effect of extracellular ATP, as conversion of adenosine to inosine, as well as of deoxyadenosine to deoxyinosine, is completely blocked, and therefore, dATP accumulates in T cells after addition of extracellular ATP. Interestingly, this was not observed in our experiments. It can be speculated that the maximal effect has already been reached without inhibition of ADA. As the blocking of ENTs and the AK completely reverted the inhibition of T cell proliferation in the presence of extracellular ATP, it seems likely that accumulation of dATP mediated by AK is responsible for the antiproliferative effect. This would explain the strong inhibitory effect of degradation products of extracellular ATP and AMP on T cell proliferation that is not observed when adenosine analogs are applied to the cultures that only target the A2aR and are not further metabolized. This furthermore explains why A2aRIs fail to revert T cell suppression mediated by ATP and AMP degradation products. This hypothesis is further strengthened by the observation that inhibition of the RNR had the same inhibitory effect on T cell proliferation as observed after addition of extracellular ATP.

Apart from inhibition of proliferation via inhibition of the RNR, dATP is known to induce cytochrome *c* release, and it is part of the apoptosome.^33^, ^34^, ^35^ Therefore, we investigated whether addition of extracellular ATP to activated human T cells would lead to an increased rate of apoptosis. In fact, ATP supplementation led to a higher percentage of apoptotic cells, further strengthening our hypothesis that accumulation of dATP might be the causative agent. Again, treatment of T cells with CD39- and/or CD73-specific ASOs, an ENT or AK inhibitor, but not with A2aRIs completely reverted this effect.

ATP and its degradation products adenosine and adenine were shown to be enriched in B cell lymphoma cell-culture supernatants and to inhibit CD8^+^ T cell proliferation.^41^ The presence of intracellular purine metabolites has not been shown in this study. Again, accumulation of dATP could be the driving force for inhibition of T cell proliferation in this setting. This is supported by the fact that the authors observed reversion of inhibitory effects of purine metabolites to a lesser extent when A2aR^−/−^ T cells had been tested as compared to CD39^−/−^ T cells.

Adenosine within the tumor microenvironment is a major mechanism of immune evasion,^47^, ^48^, ^49^ and the inhibition of adenosine receptors shows promise in preclinical models^18^, ^19^, ^20^ and in a cohort of renal cell cancer patients.^21^ However, blocking the degradation of extracellular ATP does not only prevent the generation of adenosine but additionally, leads to accumulation of extracellular ATP. Extracellular ATP activates the P_2_X_7_-NLRP3-inflammasome IL-18 pathway that increases CD8^+^ effector T cell function within the tumor microenvironment.^5^ As AMP as a substrate for CD73 enzymatic activity can also be generated by CD38, the combination of blocking CD39 and CD73 activity seems very attractive. Accumulation of immune-stimulatory extracellular ATP is achieved via CD39 blocking, whereas the combined blocking of CD39 and CD73 maximally suppresses formation of adenosine and therefore, simultaneously prevents A2aR-dependent, as well as A2aR-independent, immunosuppression exerted by downstream metabolites of adenosine. This suggestion is supported by the fact that a combination of blockade of multiple components of the adenosine axis has been reported to synergize.^15^ Here, the cotargeting of CD73 and the A2aR resulted in a more pronounced antitumor activity than the blockade of only one component. Interestingly, the tumor counteracted A2aR deficiency with increased CD73 expression. A synergistic effect of cotargeting CD73 and the A2aR has also been shown in combination with a dendritic cell-based therapeutic cancer vaccine.^40^ Therefore, the examination of coinhibition of CD39 and CD73 *in vivo* is of great interest, as *in vitro* synergistic effects with blocking antibodies could be demonstrated.^11^

In conclusion, we investigated the effects of interference with the adenosine axis at different levels in an *in vitro* human T cell system. This isolated system allowed us to decipher the effects of ATP degradation products on T cell functionality. We show here that the effect of adenosine and subsequent A2aR signaling might primarily affect cytokine secretion, whereas other purines, e.g., dATP, might especially impair proliferation of activated T cells. Therefore, inhibition of CD39 (and CD73) has distinct advantages over inhibition of the A2aR: (1) accumulation of immune-stimulatory ATP and (2) inhibition of the generation of immunosuppressive purines besides adenosine and subsequent reversion of suppression of T cell proliferation and induction of apoptosis.

## Materials and Methods

### Antisense Oligonucleotides

ASOs were selected based on the human CD39 mRNA (encoded by the *ENTPD1* gene, GenBank: NM_001776) or human CD73 mRNA (encoded by the *NT5E* gene, GenBank: NM_002526.3) using an in-house bioinformatics pipeline to ensure selectivity and avoid undesired off-target effects. LNA-modified gapmers were ordered from Exiqon (Vedbaek, Denmark) or Axolabs (Kulmbach, Germany) and dissolved in H_2_O (stock concentration: 1 mM). Sequences of ASOs and the control oligonucleotide used in the study are shown in Table 1.

**Table 1:** List of Antisense Oligonucleotides Used in This Study

ASO ID	Sequence
A04040H = CD39 ASO	+G∗+T∗+T∗T∗G∗T∗G∗T∗G∗A∗G∗A∗G∗C∗+T∗+T
A05027H = CD73 ASO	+G∗+C∗+A∗C∗T∗C∗G∗A∗C∗A∗C∗T∗T∗+G∗+G∗+T
Control oligo^24^	+C∗+G∗+T∗T∗T∗A∗G∗G∗C∗T∗A∗T∗G∗T∗A∗+C∗+T∗+T

+, LNA-modified nucleotides; ∗, phosphorothioate (PTO) linkages.

### Proliferation Assay

PBMCs were obtained from leukapheresis products (Klinikum rechts der Isar, TU München, Germany; Ethics Commission reference: 329/16 S). T cells were either isolated using CD3 Microbeads (Miltenyi Biotec, Bergisch-Gladbach, Germany) or the EasySep Human T Cell Isolation Kit (STEMCELL Technologies, Vancouver, Canada), according to the manufacturer’s instructions. T cells were washed with PBS (Thermo Fisher Scientific, Germering, Germany) and labeled with eBioscience Cell Proliferation Dye eFluor 450 (10 μM; Thermo Fisher Scientific), according to the manufacturer’s instructions. T cells (100,000 per well) were plated in 96-well U-bottom plates in RPMIfs (RPMI, 1% antimycotic-antibiotic [100×], 1% sodium pyruvate [100×], 10% heat-inactivated fetal bovine serum [FBS]; all Thermo Fisher Scientific), supplemented with 12.5 μL/mL ImmunoCult Human CD3/CD28/CD2 T Cell Activator (STEMCELL Technologies) and treated with 5 μM of ASO without using a transfection reagent. After 3 days, fresh medium was added in all experiments, and ASO was replaced in some experiments (Figures 2 and 3). ATP or AMP dissolved in H_2_O, or NECA, CGS 21680, or CADO dissolved in DMSO (all Sigma-Aldrich, Steinheim, Germany) was added at the indicated concentrations directly on day 0 (Figure 4), on day 3 (Figures 4, 6, and 7), or on days 3 and 4 (Figures 2 and 3). A2aRIs AZD-4635 and CPI-444 (MedKoo Biosciences, Morrisville, NC) dissolved in DMSO were added on days 3 and 4 (10 μM). A2bRI PSB-603 (10 μM), NBTI (20 μM), Phloridzin (20 μM), and 8-aminoguanosin (100 μM) (all Sigma-Aldrich); 9-deazaguanin (at indicated concentrations; Santa Cruz Biotechnology, Heidelberg, Germany); or COH29 (at indicated concentrations; Hölzel Diagnostika Handels, Köln, Germany) dissolved in DMSO and A-dAdo (10 μM) (Glentham Life Sciences, Corsham, UK) or 2′-deoxycoformycin (dCF; 10 μM) (Sigma-Aldrich) dissolved in H_2_O were added on day 3. On day 5, expression of CD39 and CD73, proliferation, and absolute cell numbers were analyzed by flow cytometry (see below). The proliferation index was calculated using the formula:$\sum_{0}^{i}Ni/\left(\sum_{0}^{i}Ni/2\right)$, where i is the generation number, and N is the absolute cell count in the respective generation. Cell-culture supernatants were harvested at the indicated time points, and IFN-γ concentrations were determined by enzyme-linked immunosorbent assay (ELISA).

For intracellular cytokine staining, ATP, AMP, NECA, CGS 21680, or CADO was added at the indicated concentrations on day 3. On day 5, Brefeldin A (BFA) Solution (BioLegend, Koblenz, Germany; 420601; according to the manufacturer’s instructions) was added to the cultures. Cells were cultured with BFA for 4 h and stored at 4°C until flow cytometry staining was started.

### Flow Cytometry

10,000 123count eBeads (Thermo Fisher Scientific) were added per well to be able to calculate absolute cell counts. Subsequently, T cells were spun down at 700 × *g* for 1 min and washed in fluorescence-activated cell sorting (FACS) buffer (1× PBS, 2% FBS; Thermo Fisher Scientific), followed by incubation for 30 min at 4°C in 50 μL FACS buffer per well in 96-well U-bottom plates containing the respective antibodies (anti-human CD8 allophycocyanin [APC]; Thermo Fisher Scientific; 17-0088-42, clone RPA-T81, 1:200 dilution), anti-human CD4 BV510 (BioLegend; 317444, clone OKT4, 1:200 dilution), anti-human CD39 phycoerythrin (PE)-cyanine 7 (Cy7) (BioLegend; 328212, clone A11:200 dilution), anti-human CD73 fluorescein isothiocyanate (FITC) (BioLegend; 344016, clone AD2, 1:200 dilution), anti-human adenosine A2aR PE (Santa Cruz Biotechnology; sc-32261 PE, clone 7F6-G5-A2, 1:10 dilution), anti-human CD8a BV785 (BioLegend; 301046, clone RPA-T8, 1:200 dilution), goat anti-human adenosine A2bR (Sigma-Aldrich; SAB2500030-100UG, polyclonal, 1:50 dilution), donkey anti-goat immunoglobulin G (IgG) Alexa Flour 647 (Invitrogen; A32849, polyclonal, 1:50 dilution), anti-human CD4 PE (BioLegend; 344605, clone SK3, 1:100 dilution), anti-human CD25 BV785 (BioLegend; 302638, clone BC96, 1:100 dilution), and 7-AAD viability staining solution (BioLegend; 420404, 1:50 dilution) or eBioscience Fixable Viability Dye eFluor 780 (Thermo Fisher Scientific; 65-0865-14, according to the manufacturer’s instructions). The following isotype control antibodies were used: mouse IgG1 κ PE-Cy7 (BioLegend; 400126, clone MOPC-21), mouse IgG1 κ FITC (BioLegend; 400108, clone MOPC-21), and mouse IgG2a κ PE (BioLegend; 400108, clone MOPC-173). For some experiments (Figure 1), we blocked Fc receptors using Human TruStain FcX (BioLegend; 422302, 1:50 dilution) prior to staining.

For staining of FoxP3, cells were fixed/permeabilized after surface staining using the eBioscience Foxp3/Transcription Factor Staining Buffer Set (Thermo Fisher Scientific; 00-5523-00, according to the manufacturer’s instructions), followed by incubation for 30 min at 4°C in 50 μL permeabilization buffer containing anti-human FoxP3 APC (Miltenyi Biotec; 130-113-470, clone 3G3, 1:100 dilution).

For intracellular cytokine staining, cells were fixed/permeabilized using the Cyto-Fast Fix/Perm Buffer Set (BioLegend; 426803, according to the manufacturer’s instructions), followed by incubation for 30 min at 4°C in 50 μL Cyto-Fast Perm/Wash solution containing the respective antibodies (anti-human IFN-γ APC [BioLegend; 502512, clone 4S.B3], anti-human IL-2 [BioLegend; 500304, clone MQ1-17H12], and anti-human TNF-α [BioLegend; 502929, clone Mab11], all 1:100 diluted).

Subsequently, cells were washed twice with FACS buffer and analyzed on a NovoCyte flow cytometer (ACEA Biosciences, Bremen, Germany).

For annexin V staining, cells were washed with Annexin V Binding Buffer (BioLegend; 422201) once, followed by incubation for 15 min at room temperature containing the respective antibodies (annexin V APC [BioLegend; 640941] and 7-AAD viability staining solution [BioLegend; 420404], all 1:10 diluted). Afterward, cells were washed with Annexin V Binding Buffer once and analyzed on a NovoCyte flow cytometer.

### QuantiGene mRNA Expression Analysis

Expression of CD39 and CD73 on the mRNA level was determined using the QuantiGene SinglePlex RNA Assay (QuantiGene SinglePlex Assay Kit 96-well plate format and QuantiGene Sample Processing Kit for cultured cells; Thermo Fisher Scientific), according to the manufacturer’s instructions. The following probe sets were used: human *ENTPD1* (SA-11803), human *NT5E* (SA-12473), and human *HPRT1* (SA-10030). All reagents were purchased from Affymetrix/Thermo Fisher Scientific.

### Investigation of Functionality of A2aRIs AZD-4635 and CPI-444

PBMCs (250,000 per well) were plated on 96-well U-bottom plates in RPMIfs. A2aRIs (10 μM) AZD-4635 and CPI-444 were added, followed by addition of NECA at the indicated concentrations. After incubation for 1 h at 37°C, cells were activated using 12.5 μL/mL ImmunoCult Human CD3/CD28/CD2 T Cell Activator (STEMCELL Technologies) (final concentration). Supernatants were harvested 48 h later, and IFN-γ concentration in the supernatant was determined by ELISA.

### IFN-γ ELISA

IFN-γ concentrations in cell-culture supernatants were determined using Human IFN gamma Uncoated ELISA (Thermo Fisher Scientific), according to the manufacturer’s instructions.

## Author Contributions

Conceptualization, J.F., R.K., and F.J.; Methodology, J.F. and T.T.; Software, S.M.; Investigation, J.F., R.K., M.S., and S.R.; Writing – Original Draft, J.F.; Writing – Review & Editing, R.K. and F.J.; Visualization, J.F. and R.K.; Supervision, J.F., R.K., and F.J.

## Conflicts of Interest

J.F., T.T., M.S., S.R., S.M., F.J., and R.K. are employed at Secarna Pharmaceuticals GmbH & Co. KG.

## Supplemental Information

Document S1. Figures S1–S4

Document S2. Article plus Supplemental Information
