# Supplementary material for: Preventing ATP Degradation by ASO-Mediated Knockdown of CD39 and CD73 Results in A2aR-Independent Rescue of T Cell Proliferation
Source: Mol Ther Nucleic Acids. 2020 Jun 27;21:656–69. doi: 10.1016/j.omtn.2020.06.020 (PMC7393471; doi:10.1016/j.omtn.2020.06.020)
Supplement: Document S1. Figures S1–S4 [file mmc1.pdf]

OMTN, Volume *21*

Supplemental Information

Preventing ATP Degradation by ASO-Mediated Knockdown of CD39 and CD73 Results in A2aR-Independent Rescue of T Cell Proliferation

Julia Festag, Tamara Thelemann, Monika Schell, Stefanie Raith, Sven Michel, Frank Jaschinski, and Richard Klar

Supplemental Figures

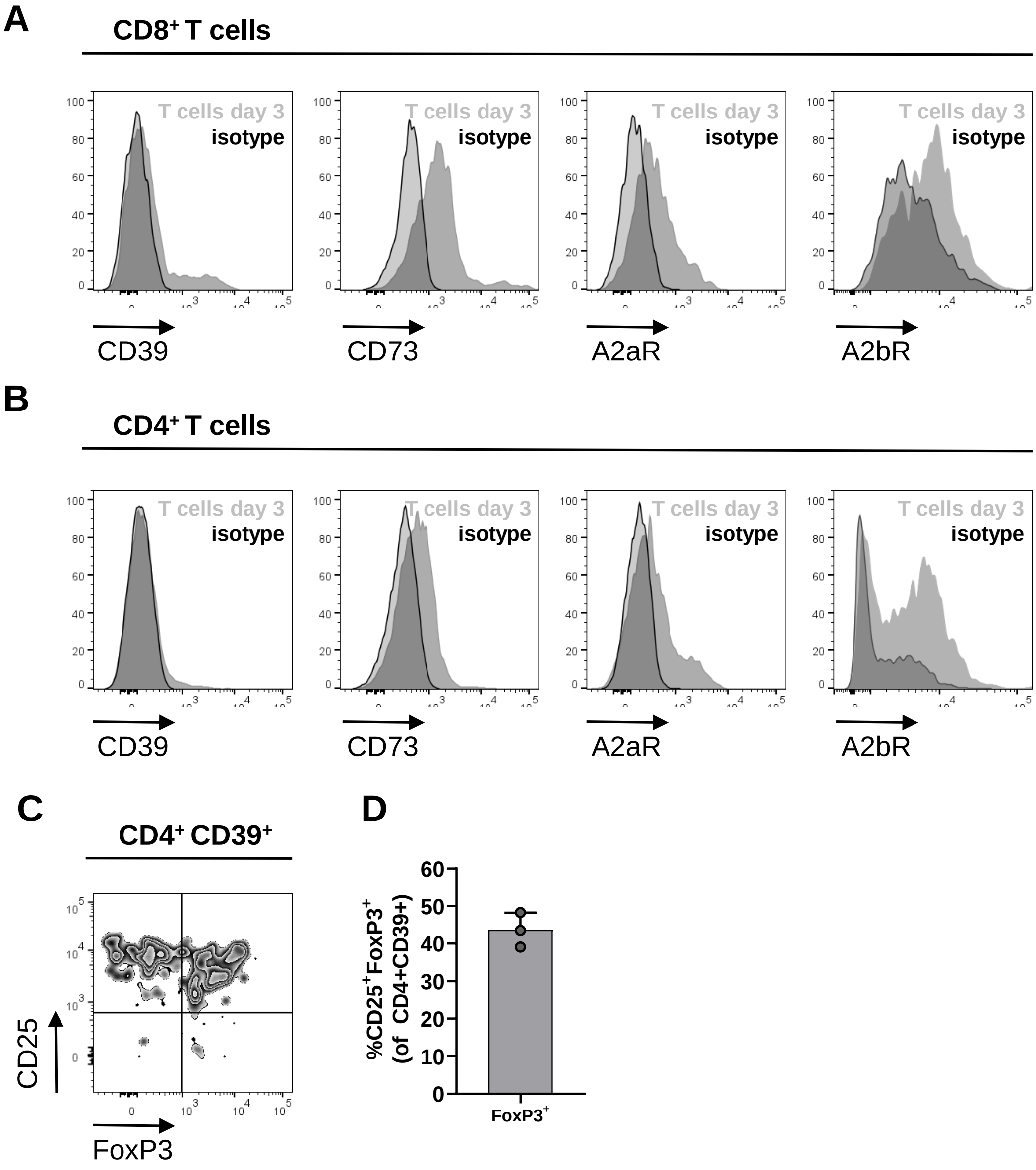

Figure S1: CD39, CD73, A2aR and A2bR expression in human T cells. CD39, CD73, A2aR and A2bR expression on day 3 after activation compared to isotype controls in CD8^{+} **(A)** and CD4^{+} **(B)** T cells. **(C)** CD25 and FoxP3 expression on CD4^{+} CD39^{+} T cells. Representative result from three technical replicates and three independent experiments are shown. **(D)** Frequency of T_{regs} (CD25^{+}FoxP3^{+}) of CD4^{+} CD39^{+} T cells. Bar graph depicts the mean of three donors run in triplicates + SD.

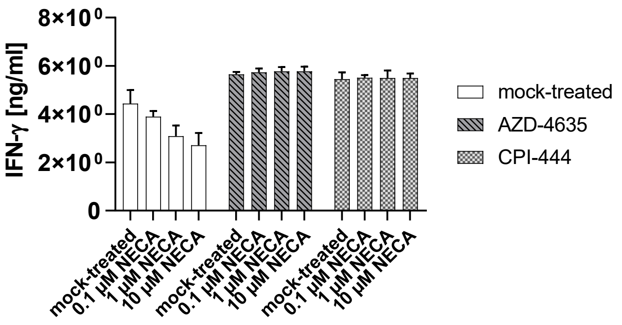

Figure S2: Effect of adenosine analogue NECA and A2aR blockade on IFN- γ secretion of activated PBMC. Human PBMC were treated with 10 µM of A2aR inhibitors AZD-4635 or CPI-444 or left untreated (mock-treated). Additionally, medium was supplemented with different concentrations of NECA. Subsequently, PBMC were activated using anti-CD2/CD3/CD28 tetrameric antibody complexes and IFN- γ concentration in the supernatant was analyzed 48 h later. The mean + SD from three technical replicates is depicted. Representative results from two independent experiments are shown.

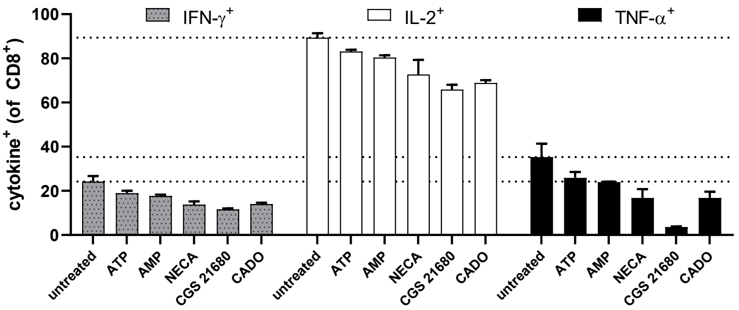

Figure S3: Pro-inflammatory cytokine secretion of T-cell cultures supplemented with extracellular ATP, AMP or the adenosine analogues NECA, CGS 21680 or CADO. Frequency of IFN-γ^+, IL-2^+ or TNF-α^+ CD8^+ T cells after addition of ATP (400 µM), AMP (600 µM) or the adenosine analogues NECA (1000 µM), CGS 21680 (1000 µM) or CADO (30 µM) on day 3 after activation. Intracellular cytokine staining was performed on day 5 after activation. The mean + SD of three technical replicates is depicted. Representative results from three independent experiments are shown.

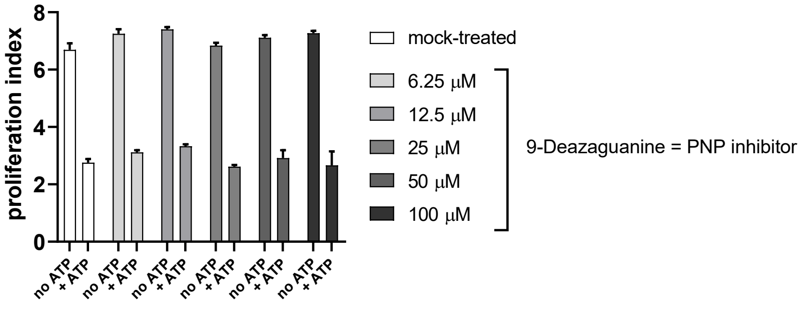

Figure S4: Influence of inhibition of PNP by 9-Deazaguanine. Human T cells were labelled with a proliferation dye and activated with anti-CD2/CD3/CD28 tetrameric antibody complexes. On day 3 PNP inhibitor 9-Deazaguanin was added at indicated concentrations and 400 µM ATP was added to the cells. Proliferation of T cells was analyzed by flow cytometry on day 5 and proliferation indices were calculated. The mean + SD of three technical replicates is depicted. Representative results from two independent experiments are shown.
